# 

**Published:** 1878-05-20

**Authors:** 


ORIGINAL AND SELECTED ARTICLES.

On Some of the Uses of Hyposulphite of Soda in Medicine, by T. B. Greenley, M.D., Ky. .... 129
Painter's Colic (Colica Pictonum), by O. E. Newton, M.D., of Cincinnati .... 130
Antagonism of Therapeutic Agents .... 131
Duality of the Brain, Read before the Meigs Co. Medical Society, by J. Barr Smith, M.D. .... 134
Gonorrhœa—Its Bathology and Treatment, by C. C. Goodshaw, A.B., M.D., read before the Louisville Academy of Medicine, April 19, '78 .... 136
Case of Iodic Purpura, by Robert Abbe, M. D., of New York .... 138
Hydrobromic Acid in Cerebro Spinal Meningitis .... 138
Hypophospite of Zinc .... 140
Society Reports—Atlanta Medico-Chirurgical Association .... 140
Medical Association of Georgia .... 142

ABSTRACTS AND GLEANINGS.

Excessive Venery, 145; Treatment of Excessive Venery, 145; Impotence, 145; Remarks on Obstetrical Operations, 146; Hydrobromic Acid in Cerebro Spinal Meningitis, 147; Chloride of Zinc in the Treatment of Ulcers of the Leg, 148; Retained Placenta in Abortion, 149; Trephining for Compression, 150; Arsenic in the Treatment of Malignant Tumors, 151; Chloral by Enema, 151; Koumiss, Glycerine and Creosote in Consumption, 152; Polyuria Successfully Treated by Ergot of Rye, 152; Absorption of Tincture of Iodine by the Skin; Viburnum Prunifolium, 153; The Latest Crucernihg Germs, 153; Boracic Acid in Skin Diseases, 153; The Sense of Smell, 153; A Remedy for the Eruption of Poison Oak, Ivy and Sumach, 154; Rapid Expulsion of Tape Worm, 154; Hœmoptysis, 154; Instantaneous Cure of Hydrocele, 154; Diarrhœa in Infants, 155; Arsenic and its Preparations in the Treatment of Skin Diseases, 155; Bismuth in the Treatment of Prolapse of the Rectum and Hemorrhoids, 155; Fibroid Polypus of the Vagina, 155.

PRACTICAL NOTES AND FORMULÆ.

Diarrhœa, 156; Hyposulphite of Soda, 156; Quartan Intermittents, 156; Night Sweats, 156; Saline Aperient, 157; Approximate Measurements, 157; Whooping Cough, 157; Weatherly's Famous Catarrh Cure, 157; Penthorum Sedoides, 157; Ergotine Hypodermically, 157; Corrosive Sublimate Hypodermically, 157; Atropia Hydodermically, 157; Nocturnal Pains, 157; Purgative Powder, 157; Subcutaneous Medication, 157; Neuralgic Remedy, 158; Arsenic in Mania, 158; Creosote in Ulcers, 158; Anti Insects, 158; Scalp wounds, 158; Valerianated Syrup of Rhubarb, 158; New Adhosive Plaster, 158; Strychnia, 158.

EDITORIAL AND MISCELLANEOUS.

Disaffection in the State Medical Association of Georgia, 159; State Medical Society of Arkansas, 160; American Medical Association, 160; Medical Graduates, 160.

